# Visualizing the Unseen: Illustrating and Documenting Phantom Limb Sensations and Phantom Limb Pain With C.A.L.A.

**DOI:** 10.3389/fresc.2022.806114

**Published:** 2022-02-09

**Authors:** Michael Bressler, Joachim Merk, Johannes Heinzel, Martin V. Butz, Adrien Daigeler, Jonas Kolbenschlag, Cosima Prahm

**Affiliations:** ^1^Department of Hand, Plastic, Reconstructive and Burn Surgery, BG Trauma Clinic, University of Tuebingen, Tuebingen, Germany; ^2^Neuro-Cognitive Modeling Group, Department of Computer Science and Department of Psychology, Faculty of Science, Eberhard Karls University, Tuebingen, Germany

**Keywords:** limb amputation, phantom limb sensation, phantom limb pain, body image visualization, altered body image, documentation methodology, digital assessment, software tool

## Abstract

Currently, there is neither a standardized mode for the documentation of phantom sensations and phantom limb pain, nor for their visualization as perceived by patients. We have therefore created a tool that allows for both, as well as for the quantification of the patient's visible and invisible body image. A first version provides the principal functions: (1) Adapting a 3D avatar for self-identification of the patient; (2) modeling the shape of the phantom limb; (3) adjusting the position of the phantom limb; (4) drawing pain and cramps directly onto the avatar; and (5) quantifying their respective intensities. Our tool (C.A.L.A.) was evaluated with 33 occupational therapists, physiotherapists, and other medical staff. Participants were presented with two cases in which the appearance and the position of the phantom had to be modeled and pain and cramps had to be drawn. The usability of the software was evaluated using the System Usability Scale and its functional range was evaluated using a self-developed questionnaire and semi-structured interview. In addition, our tool was evaluated on 22 patients with limb amputations. For each patient, body image as well as phantom sensation and pain were modeled to evaluate the software's functional scope. The accuracy of the created body image was evaluated using a self-developed questionnaire and semi-structured interview. Additionally, pain sensation was assessed using the SF-McGill Pain Questionnaire. The System Usability Scale reached a level of 81%, indicating high usability. Observing the participants, though, identified several operational difficulties. While the provided functions were considered useful by most participants, the semi-structured interviews revealed the need for an improved pain documentation component. In conclusion, our tool allows for an accurate visualization of phantom limbs and phantom limb sensations. It can be used as both a descriptive and quantitative documentation tool for analyzing and monitoring phantom limbs. Thus, it can help to bridge the gap between the therapist's conception and the patient's perception. Based on the collected requirements, an improved version with extended functionality will be developed.

## Introduction

After the amputation of a limb, up to 90% of the patients report a feeling of the missing body part still being present (1). This effect is known as phantom limb sensation (PLS) and ranges from the simple feeling of presence to the perception of a specific posture, shape, or involuntary movements of the amputated limb (2–4). Additionally to PLS, which is defined as any sensation except pain (3), 45–85% of all patients suffer from phantom limb pain (PLP), which can manifest itself as e.g., stabbing, burning, twisting, or cramping (5). The term “phantom pain syndrome” was coined by Weir Mitchell in 1871 (4) when the use of the word “phantom” was commonly used in the medical field to describe pseudo-diseases, which may have contributed to the fact that PLP was stigmatized as ”imaginary“ for a long time (6).

PLP usually manifests itself 24 h to 1 week after amputation and decreases in intensity and frequency over time in most patients (3). Especially in the distal areas of the missing limb, PLP as well as PLS generally persist the longest. Some patients suffer from this pain for decades (2, 7). The underlying mechanisms causing PLP and PLS are still discussed controversially. The current dominant theory is the cortical remapping theory, according to which the brain responds to the loss of a limb with the reorganization of somatosensory maps: cortical areas that have received sensory signals from the amputated limb begin to receive input from neighboring areas (2, 4). Another explanation is based on the concept of a “neuromatrix”—an internal representation of one's own body. After an amputation, this representation remains intact and no longer matches the actual body, thus causing pain. The absence of visual and sensitive feedback of the missing limb enhances this effect (8).

PLP, defined as painful sensation in the missing part of the limb, is to be distinguished from pain in the residual limb (9), and in particular from neuroma pain. Painful neuromas develop at the stump of the severed nerve due to misguided attempts of nerve regeneration and are one of the main causes of residual limb pain (4, 10). Physical stimulation of the neuroma in form of pressure or stress on the limb can increase PLP, and in the past, neuromas were considered to contribute to the development and maintenance of PLP. However, PLP does also occur in the absence of stump pain, and removal of a neuroma does not cause PLP to disappear (2, 3).

PLP is an elusive entity, which makes it hard to track the progress of these patients over the course of treatment. Currently, there is no standardized mode of documenting PLP and PLS. The guidelines of the German Society of Neurology for the diagnosis of neuropathic pain recommend to document the onset and duration, the temporal course, pain qualities, localization and intensity as well as factors triggering pain (11). In general, it has become common practice to survey phantom pain with pain questionnaires. For example, the McGill Pain Questionnaire (MPQ) (12) became a de facto standard for the qualitative characterization of PLP, which is reflected by the terminology used in the medical literature after 1975 (13). Other pain questionnaires, such as the Brief Pain Inventory (BPI) (14), allow for the localization of pain by marking the appropriate areas on a 2D body chart. However, this type of documentation has the disadvantage of being not very precise. Shaballout et al. showed that a digital solution for drawing pain can not only contribute to a better understanding of the pain situation for physicians, but also facilitate analysis and quantification (15). Further improvement in the precision of this approach could be achieved by drawing pain directly on a 3D model (16).

This still does not allow for the illustration of the patients' altered body image, in particular the phantom. Although several software tools do exist that can be used to illustrate an altered body image, these have been developed primarily in the context of eating disorders (17–19). Therefore, the specific representation of a phantom limb is not possible with this approach. Appropriate illustrations would require an artist guided by the patient or could be drawn by patients with the appropriate drawing or photo editing skills (20). However, this is costly and totally unfeasible in a clinical context. Furthermore, it does not allow for a quantifiable analysis.

Since we could not find any suitable software, we decided to develop such a tool ourselves. In the present study we describe the functionality of the first version of C.A.L.A. (Computer Assisted Limb Assessment) and the results of its evaluation with therapists and patients in terms of usability and functionality.

## Materials and Methods

### C.A.L.A.

The basic idea of C.A.L.A. is the customization of a virtual human 3D avatar in such a way that it represents the patient's body image including their PLS. A prototype (21) and a first version of C.A.L.A. were created by modifying and expanding the Open Source software applications MakeHuman (22), a software tool for 3D character creation, and the 3D modeling software Blender (23). This first version provided a 3D avatar that could be freely rotated and viewed from all sides and the principal functions of C.A.L.A.: (1) General adjustment of the 3D Avatar; (2) altering the shape of the phantom limb; (3) positioning the phantom limb; (4) drawing pain and cramps; and (5) the quantification of the created body image.

The process of documenting a patient over the course of treatment was as follows: Initially, a basic model is created by adjusting the 3D avatar to fit the patient's (perceived) body dimensions. This model then serves as a baseline to be built on in the following sessions. Over the course of treatment, the phantom limb can then be adjusted in terms of deformation, position, and pain, thus visualizing the changes in perception by the patient.

These functions are explained in detail in the following:

#### Adjusting the 3D Avatar

To increase the patient's identification with the 3D avatar, we used some of the original functions provided by MakeHuman, which allow for the adjustment of the avatar in terms of gender, age, muscles, weight, and proportions. These adjustments have no further purpose in the documentation process apart from cosmetic ones. The avatar can additionally be clothed with underwear.

#### Measuring the Patient

The patient's body measurements can be transferred to the avatar. The body height as well as circumference and length of upper arm, forearm, upper and lower leg, fingers, and toes as well as the length and width of hands and feet can be entered and form the basis for the subsequent measurements of the phantom limb.

#### Modeling the Phantom Limb

**Figure 1:**
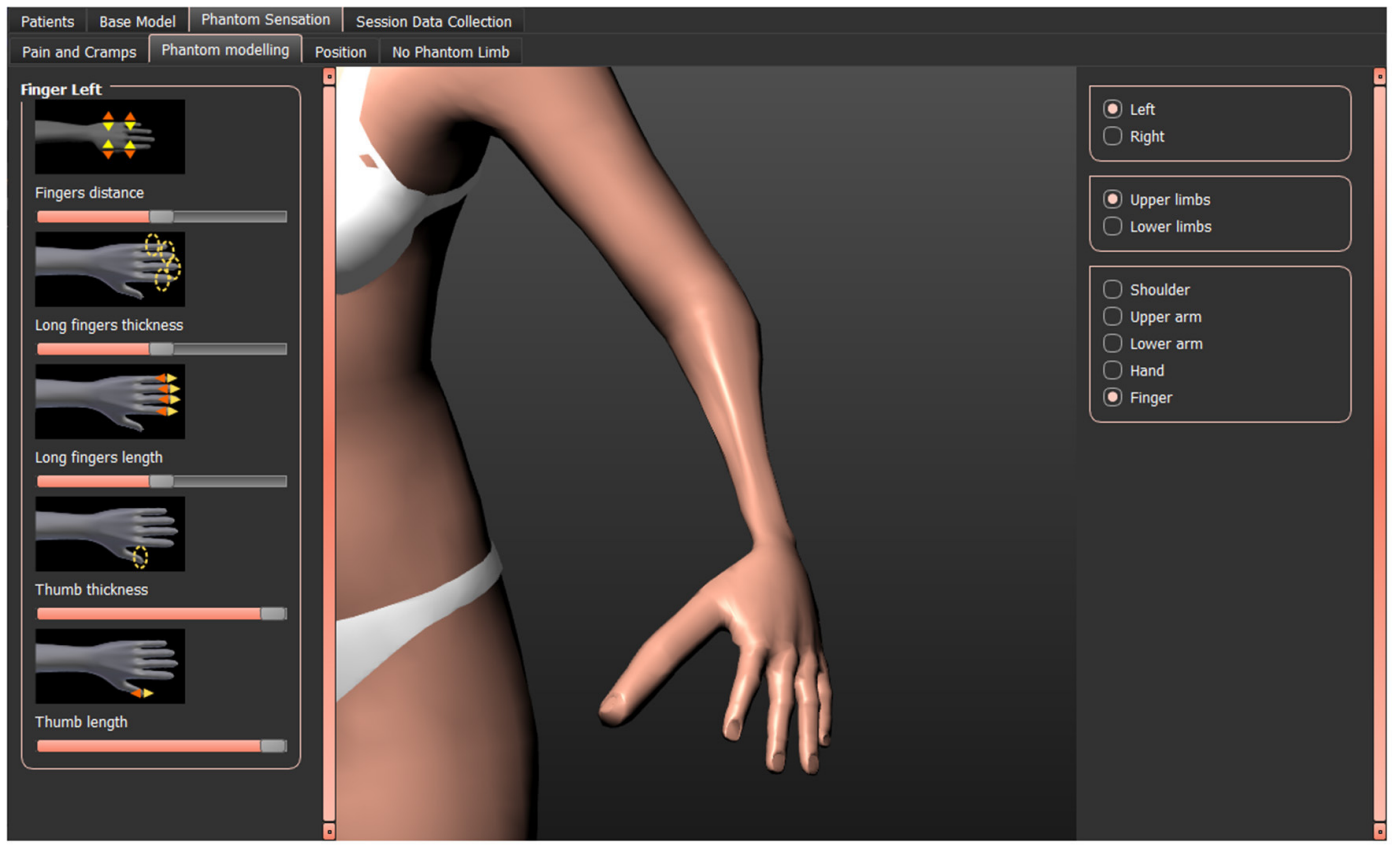
Adjusting the shape of the phantom limb by decreasing the thickness of the lower arm and increasing length and thickness of the thumb.

The length and circumference of the upper arm, forearm, thigh, and lower leg can be increased or decreased. Hands and feet can be enlarged or shrunk. Fingers and toes can be adjusted in length and circumference, the thumb and long fingers can be adjusted separately. The telescoping effect can be represented using this feature (see Figure 1).

#### Positioning the Phantom Limb

**Figure 2:**
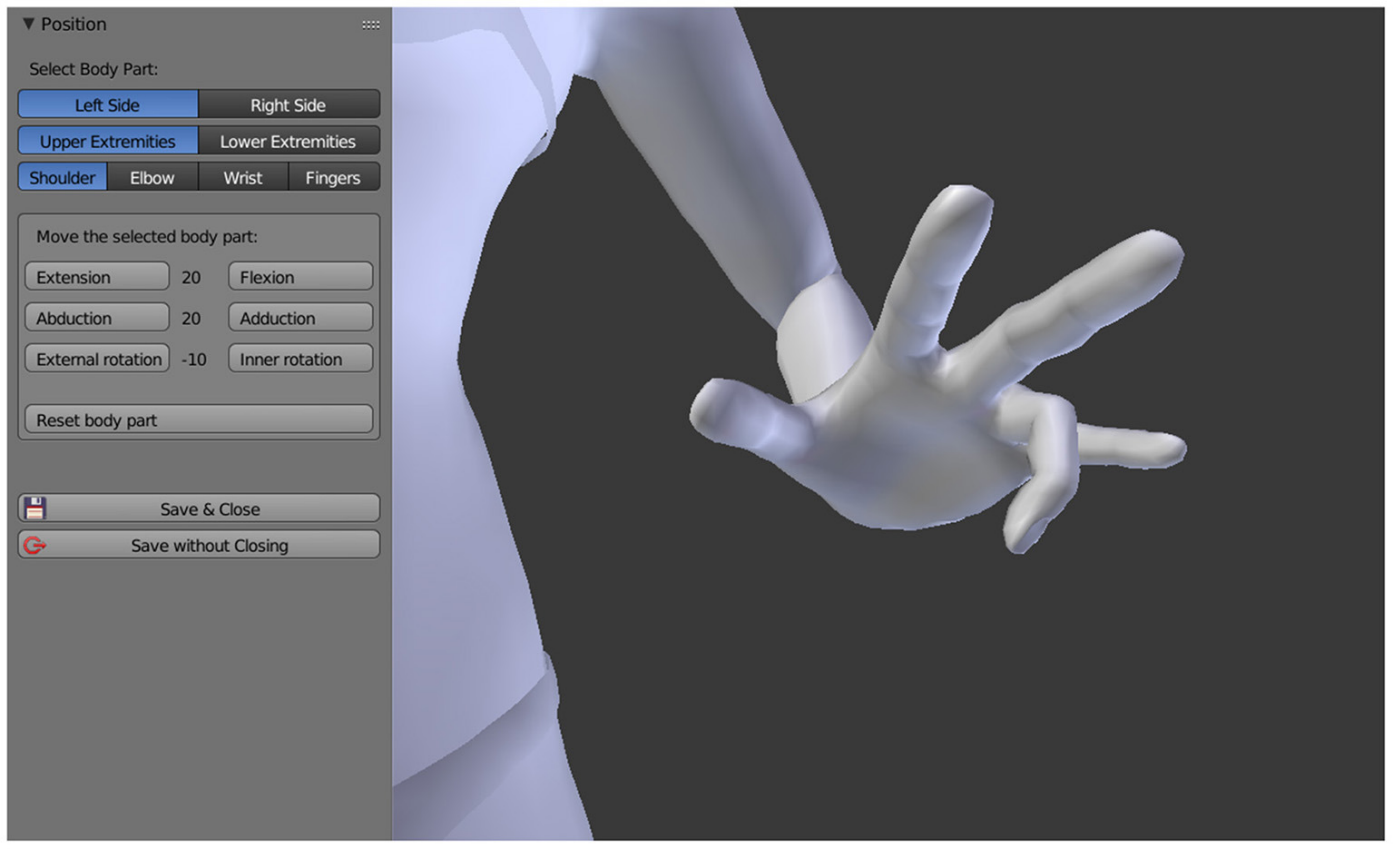
Adjusting the position of the phantom limb by rotating the respective joint axes.

The sensation of the phantom limb being fixed in one or more, twisted or unnatural positions is captured by moving the respective joints of the 3D avatar into the position reported by the patient. Based on the original MakeHuman 3D model, it is possible to rotate the shoulder, elbow, and wrist joints as well as the individual finger joints of the 3D avatar, the same applies to the joints of the lower extremities. All joints can be rotated along their natural axes in steps of ±10° and even beyond the limits that are anatomically possible. As a result, all conceivable positions of the upper and lower extremities can be represented (see Figure 2).

#### Drawing Pain and Cramps

**Figure 3:**
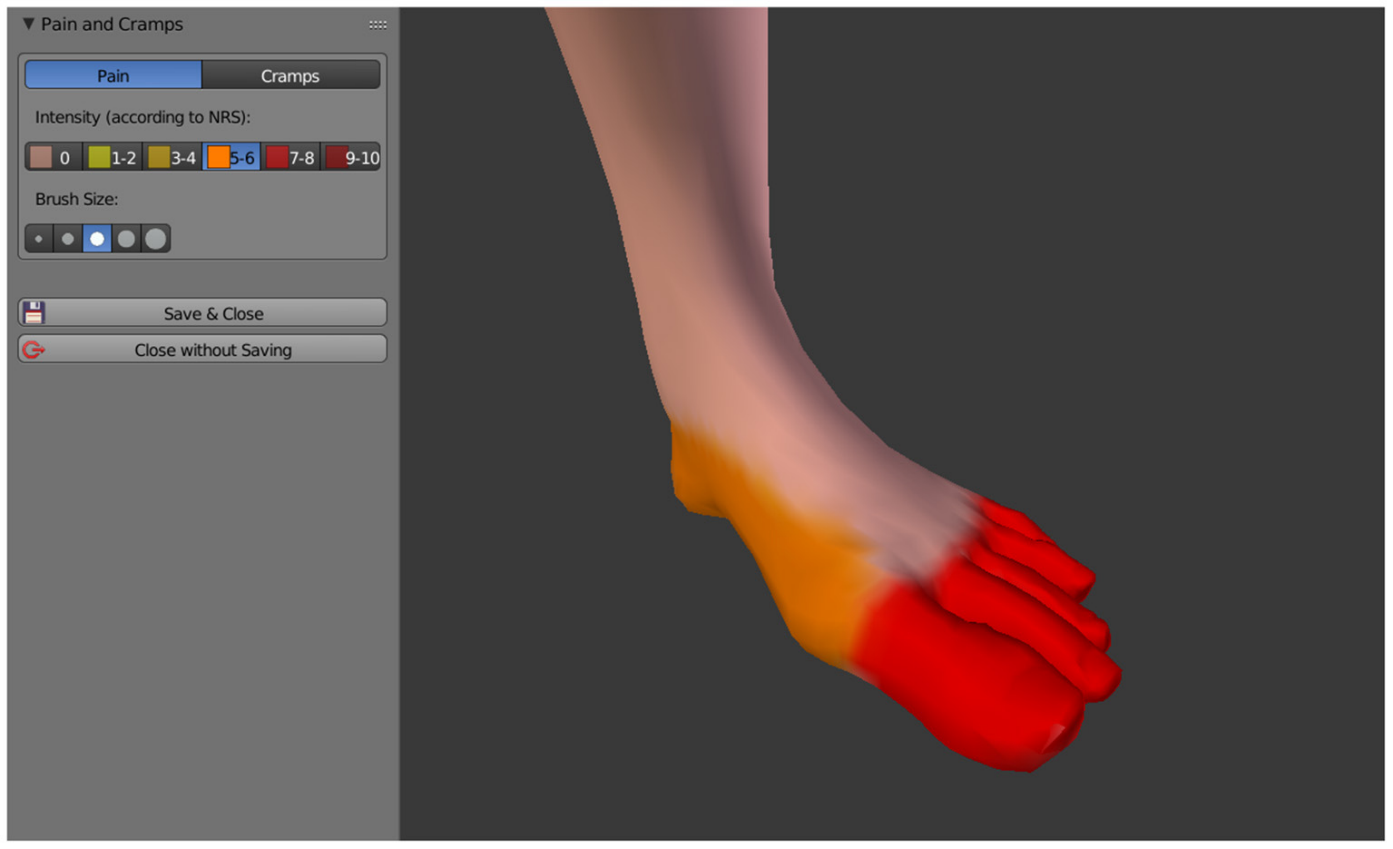
Pain drawn directly onto the 3D avatar in different intensities.

Pain is drawn directly onto the 3D avatar by using the mouse cursor as a brush, similar as it is done in 2D paint software. Currently C.A.L.A. distinguishes between pain in general and cramps in the phantom, these two aspects can be drawn independently of each other and with their respective intensity (see Figure 3), which is indicated by the Numeric Rating Scale (NRS) with a value between 0 and 10. The intensity is represented by different color schemes, general pain by a color gradient from yellow (slight pain) to dark red (severe pain), cramps by a color gradient from light blue (slight cramping) to dark blue (severe cramping).

#### Quantifying the Body Image

All data that were entered during the documentation process can be quantified and contain informative value about the phantom's constitution at the respective time. This allows for the analysis of the recorded aspects, namely deformation, position, and pain, and for their observation over the course of treatment. The quantification of these three aspects is briefly described as follows: Quantification of deformation reflects the percentage change in length and circumference of the respective limbs compared to the base model. Based on the originally collected dimensions of the patient's body, these changes can also be expressed absolutely in centimeters. The quantification of the position results from the deviation of each rotation axis of each joint from the basic position of the 3D avatar. Pain and cramps are quantified as the percentage of the body surface that is covered by the respective intensity.

### Participants: Evaluation With Therapists

C.A.L.A. was evaluated with 33 professionals (19 physical therapists, 9 occupational therapists, 2 orthopedic technicians, 3 medical staff). Of these, 22 were female and 11 were male with an age range of 25–58 and a mean age of 41. The inclusion criteria for all participants were to actively work with amputees and document their phantom limb in a clinical context.

Each participant was initially provided with a brief introduction to the operation of C.A.L.A. Subsequently, participants were given the task to perform the entire documentation process (see Section C.A.L.A.) on two given, fictional patients (see Supplementary Material). These tasks were the same for all participants. It included the creation of a basic model, adjustment of the phantom's deformation, adjustment of the phantom's position, and finally drawing pain and spasms. All participants were observed while performing the tasks and provided with assistance in operating the software. The duration for completing each task was measured and the level of assistance required was rated on a 1–5 Likert Scale by the investigator.

Subsequently, all participants were questioned with the System Usability Scale (24) to determine the user-friendliness of the software. With an additional self-developed questionnaire (see Supplementary Material) and semi-structured interview, the therapist's methods of documenting phantom pain and phantom sensation were surveyed and the principal functions of C.A.L.A. were rated. In the semi-structured interview, difficulties regarding the use of C.A.L.A., suggestions for improvement and additional desired functionalities as well as application scenarios were collected.

### Participants: Evaluation on Patients

To test the scope of the currently implemented functionality regarding real-world cases of PLS and PLP, we evaluated C.A.L.A. on 22 patients with the following amputations: 1 × transhumeral, 1 × transradial, 12 × transfemoral, and 5 × transtibial, thereof one patient with a transfemoral and one with a transtibial amputation of both legs, 3 × finger amputation. Eight of the patients were female and 14 were male with an age range of 21–73 and a mean age of 52. The inclusion criterion for all patients was the amputation of at least one limb.

For each patient, the entire C.A.L.A. documentation process was performed (see Section C.A.L.A.) by the investigators. The therapists who took part in our study did not evaluate the patients. Subsequently, the patients were questioned about their phantom pain with the German version of the Short Form McGill Pain Questionnaire (SF-MPQ-D) (25) to assess the presence of the different pain qualities. We administered a self-developed questionnaire (see Supplementary Material) with a 1–5 Likert scale rating system (“very inaccurate” to “very accurate”) to determine how accurately the patients rated the representation of deformation, position, and pain of their phantom, and which aspects could not be mapped.

## Results

### Evaluation With Therapists

**Figure 4:**
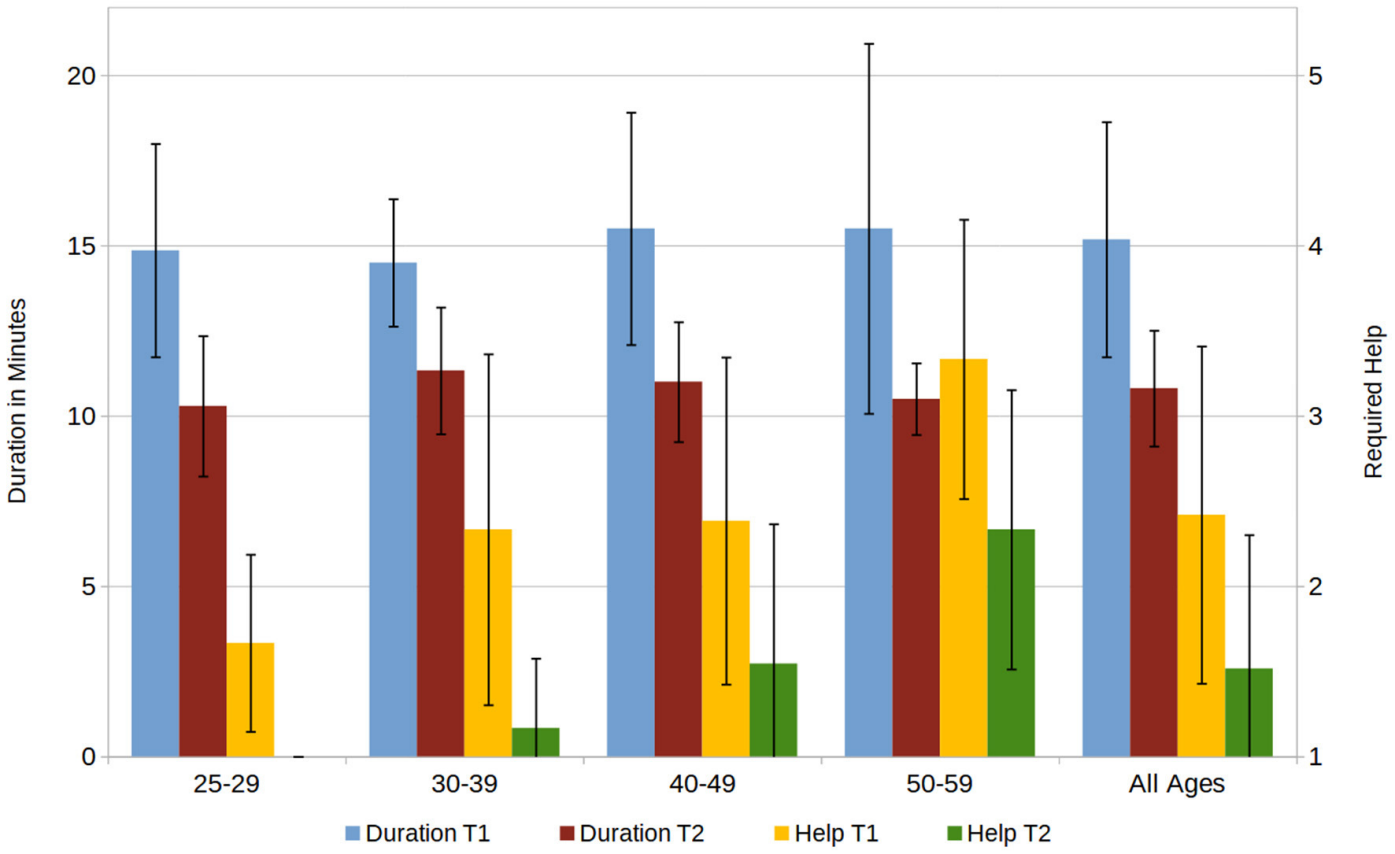
Required duration for completion of the first and second task (T1, T2) in minutes and the required assistance, rated on a 1–5 Scale (1 = “no help,” 5 = “a lot of help”).

All 33 participants completed the documentations of two given fictional patients. The average duration needed to complete a task decreased from 15.2 (±3.5) min for the first task (T1) to 10.8 (±1.7) min for the second (T2), the assistance provided by the investigator, measured on a 1–5 Likert scale (“very little help” to “very much help”), decreased from 2.4 (±1.0) to 1.5 (±0.8). Broken down by age group, the duration was very similar through all groups, however the amount of help provided was the highest for the oldest age group and the lowest for the youngest age group (see Figure 4).

The evaluation with the System Usability Scale resulted in an average score of 81.7% (±11.2), placing in the 4th quartile which represents high usability. The values are similar across age groups and professions. Additionally, we evaluated the usability of C.A.L.A. by user observation and semi-structured interviews, in which we asked about the difficulties in using C.A.L.A. Several users mentioned that the controls were too small and too cluttered. We also observed operational errors (such as modifying the wrong side of the body), problems understanding the user interface and difficulties navigating the 3D avatar.

With a separate, self-developed questionnaire and semi-structured interview we prompted the participants about their own documentation methods during therapy. Regarding the use of templates or specific questionnaires, 45% of the participants reported to use body charts to draw pain and 27% use validated questionnaires to assess pain, PLS, or body image. Besides questionnaires, the documentation was usually mostly handwritten and in a self-defined form.

**Figure 5:**
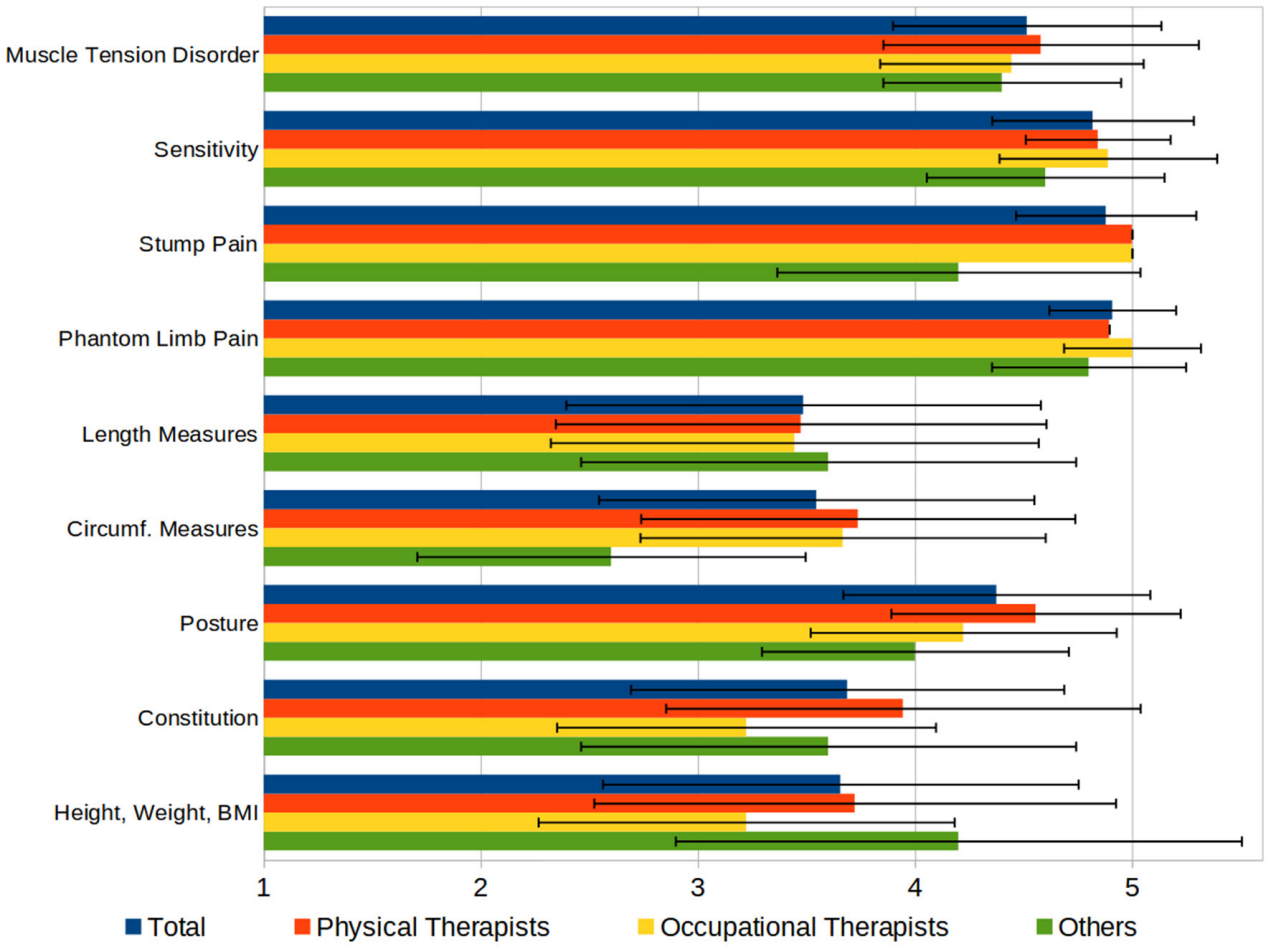
Aspects of a therapeutic finding, rated by importance on a 1–5 Scale (1 = “not important,” 5 = “very important”).

We asked the participants to rate various aspects of the therapeutic finding by their importance on a scale from 1 to 5 (“not important” to “very important”). The results (see Figure 5) show the high importance of pain, sensation, and muscle tension, compared to the measures of the patient's body or their physical condition.

**Figure 6:**
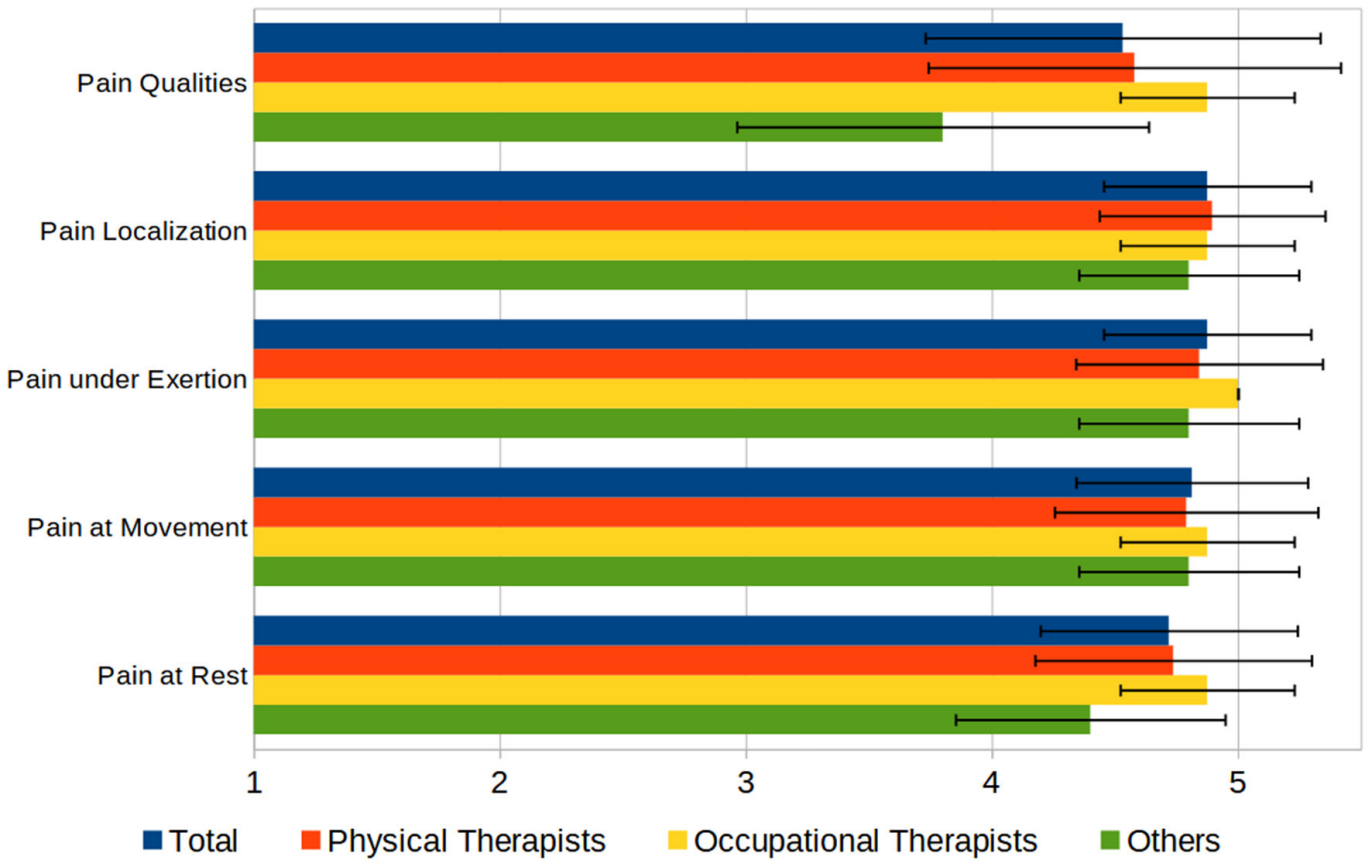
Aspects of documenting pain, rated by importance on a 1–5 Scale (1 = “not important,” 5 = “very important”).

Regarding the documentation of pain, most of the participants (76%) used the Numeric Rating Scale (NRS) and 51% used the Visual Analog Scale (VAS) (26) to assess the intensity of pain. Twenty-four percent of the participants used pain questionnaires, with no questionnaire being reported more than once. Other aspects of pain such as influencing factors (e.g., medication, psychological state), temporal (24-h) course, and duration are documented in a free form. Questioned about the importance of several aspects of pain in documenting rated on a 1–5 scale (“not important” to “very important”) showed that in average all aspects have been rated above 4.5 (see Figure 6).

**Figure 7:**
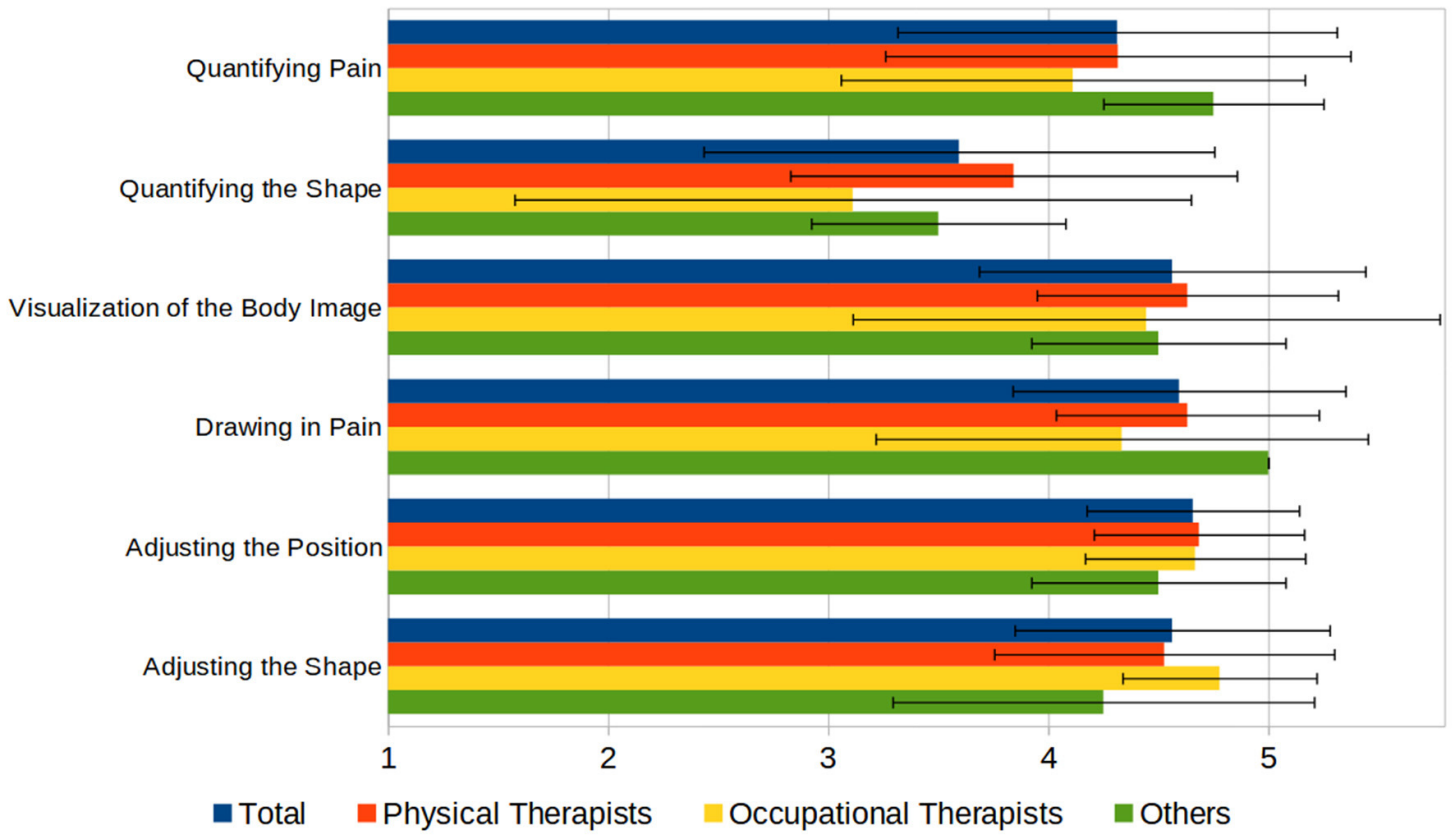
Principal functions of C.A.L.A., rated by usefulness on a 1–5 scale (1 = “not useful,” 5 = “very useful”).

Subsequently, we asked the participants to evaluate the functionality of the C.A.L.A. features. Using a Likert scale from 1 to 5 (“very low” to “very high”), participants were asked to rate the usability of the functionalities regarding the documentation of phantom limbs on a 1–5 scale (“not helpful” to “very helpful”), which resulted in high acceptance of the functions, rated least was the function to quantify the deformation of the phantom with 3.6 (±1.2) (see Figure 7).

In the semi-structured interview, we asked about additional functionalities for C.A.L.A., the most frequently mentioned ones are listed as follows: The documentation of pain qualities and the temporal aspects of pain (course, duration, frequency) were mentioned very frequently, not only in relation to phantom pain but also to residual limb pain. Another request concerned the modeling and positioning of the body parts, here a more differentiated adjustment, especially of the fingers and toes, was asked for. Regarding the positioning of the phantom, it was suggested to use a standardized diagnostic specification to describe the rotation of the joints, e.g., the deviation from the neutral zero position (27).

**Figure 8:**
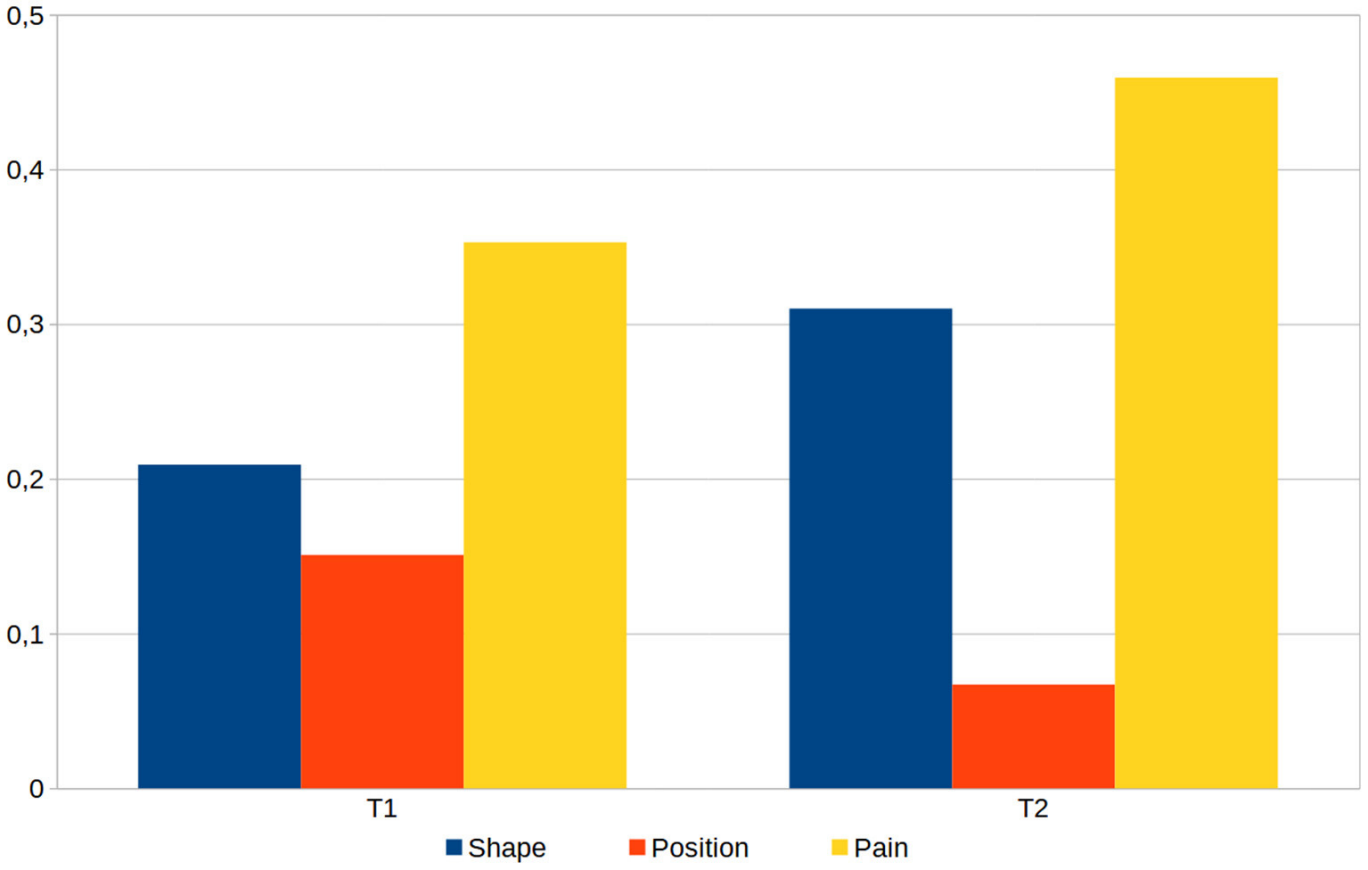
The averaged coefficient of variance calculated for the three main aspects shape, position and pain, as determined from all 33 generated models for task 1 (T1) and task 2 (T2) of the therapist evaluation.

Finally, we compared the quantifiable aspects of the documentations that have been created in the first respective second task by all participants, namely the shape and position of the phantom, and the drawing of pain and cramps. Analyzing the data revealed that during the documentation of both tasks, 3—always different—participants mixed up the side of the body and worked on the wrong arm or foot. The data of these six documentations were corrected by the side of the body and added to the evaluation. We determined the coefficient of variance for all parameters of the respective documentation aspects (deformation, position, and pain) and calculated their mean values (see Figure 8). This shows the smallest deviations when setting the position and the largest deviations when drawing pain, which is true for both tasks.

### Evaluation on Patients

Seventeen of the 22 patients were experiencing PLP, five of them reported a deformed phantom and five reported a twisted position of the phantom. Thirteen patients reported suffering from stump pain.

All 17 patients with PLP or a deformed or twisted phantom were asked how well their phantom and their body image could be mapped, rated on a 1–5 Likert scale (“very inaccurate” to “very accurate”). The other five patients, who only reported stump pain were just asked about the accuracy of their body image representation. The phantom was rated with an average of 4.6 (±0.7), the body image with 4.2 (±1.0). We also asked the patients, how important the aspects of gender, age, and physical shape were for them regarding the accurate representation of body image, which revealed that these aspects were not of primary concern.

Regarding the functional range, it was often remarked that the body image was inaccurate due to the missing visualization of the residual limb. Modeling of the individual fingers was required in greater detail than provided, both in terms of deformation and position. It was also not possible to visualize, that some parts of the amputated limb were still present as phantom sensation while other parts were no longer perceived.

The documentation of pain revealed the missing option of documenting different qualities of pain. Here, especially the pain quality “stabbing” was mentioned several times. Another functional absence was the description of the temporal aspects of pain, such as long, short, or periodic pain. In addition, patients mentioned various other aspects when describing their pain, such as the course of the pain experience, the time of day, whether the patient was resting or moving, or even the influence of weather.

Subsequently, all 22 patients were interviewed with the SF-MPQ-D to measure number of pain qualities mentioned per patient and the frequency of each pain quality. For the patients without phantom pain, stump pain was queried instead. On average, 4.8 (±2.8) of 15 qualities were mentioned per patient, the most frequently mentioned were “shooting,” “stabbing” and “hurting.” During the interview as well as during the documentation of pain in C.A.L.A., it became apparent that the distinction between stump and phantom pain was not clear for many patients and therefore a mixture of both pain sensations was sometimes described.

## Discussion

### Advantages of C.A.L.A.

The evaluation of C.A.L.A. with the System Usability Scale and the survey have shown that the vast majority of the participants considered C.A.L.A. user-friendly and feasible. To our knowledge, there is currently no software tool for therapists that allows for the visualization of phantom limbs, especially considering deformation, position, and pain. Therefore, rendering a comparison of C.A.L.A. to any existing standard regarding the documentation of phantom limbs is practically impossible. In a clinical setting time is of the essence. The duration of the documentation averages at 10 min, reducing the time by about one third in the second documentation trial, indicating that more training will likely reduce the time further.

During the evaluation with patients, they reported of never having given this amount of thought to the exact nature of their phantom limb. This fact was especially observable in localizing PLP. It was stated in only one case that the process of visualizing the phantom had a negative impact on the patient's body image. No patient indicated that phantom pain had increased because of the documentation process.

In this study we emphasized validity and did not specifically test for reliability, due to the nature of the modeling and positioning of the phantom limb and pain, which is dependent on the accuracy of the patient's report. We have provided different levels of detail in the tasks for position, deformation and pain, which is supported by the documentation differences shown in Figure 8. Especially regarding pain, room was intentionally left for interpretation, mimicking actual interactions with patients. In doing so, pain drawing could be assessed which resulted in the high variance. Corrective interventions during the dialogue with the patient could have lowered the outcome in variance.

Since all body image data are available in digital form, they can be easily quantified. This allows for a much more precise and simpler quantification than it would be possible with the conventional, mainly analog, methods. The amount of pain drawn onto a 2D human outline as well as joint angles of the phantom could possibly be estimated as could the circumference and the length. However, to our knowledge no one has ever calculated such values, especially regarding position and deformation, nor have their changes been evaluated over the course of treatment. To the best of our knowledge, no tools exist, yet, which can be used to document phantom limbs and PLP. C.A.L.A. offers a convenient tool to document just that.

In addition to evaluating usability, an essential aspect of our study was to identify possible extensions and adaptations of the functional scope. These will be discussed as follows.

### The Struggle With Documenting Pain

When documenting PLP and PLS, pain is clearly the most important issue. Pain affects the patients' quality of life, and its reduction usually is the primary goal of therapy. The importance of pain was also evident in the qualitative surveys with patients, in which it was described by far the most frequently and in the greatest detail. In the therapist survey, too, there was the most feedback on the topic of pain documentation.

In this context, the topic of pain qualities was most frequently mentioned by both patients and therapists. This is not surprising since using these pain qualities for describing PLP had been established almost 50 years ago (13). Currently, in C.A.L.A. it is only possible to enter “general” pain and the pain quality “cramping.” Expanding this to document other pain qualities seems useful, whereby clustering them to a few 5–10 qualities would be necessary. The current method of evaluating the pain intensity using the NRS is a common approach among the interviewed therapists (used by 76%).

In addition to the localization, intensity and the qualities of pain, the guidelines of the German Society of Neurology (11) recommend documenting the aspects of duration and temporal course as well as the factors that trigger pain. In addition, the qualitative evaluation also revealed quite a few other aspects of pain relevant for a complete description, e.g., deep/superficial pain. However, all mentioned aspects have in common that their visualization in C.A.L.A. would be difficult and not very intuitive to understand. We therefore consider it useful to omit these aspects from the documentation of PLP in C.A.L.A.

### Representation of Phantom and Stump

Besides the issue of pain, C.A.L.A. should include means of clearly visualizing the stump to make it easier to distinguish it from the phantom. Several patients stated during the qualitative interviews that the visualization of their body image was not complete due to the missing visualization of the stump, even if the sensation of the phantom limb was present in the patients.

When drawing PLP based on the patients' descriptions, it became obvious that the strict distinction between phantom pain (exclusively in the missing part of the limb) and stump pain (exclusively in the part still present) (9) was not necessarily useful for the patients. Although we pointed out that we intended to document only phantom pain, in some cases the pain described extended from the phantom to the existing limb, in a few cases even to the middle of the body.

**Figure 9:**
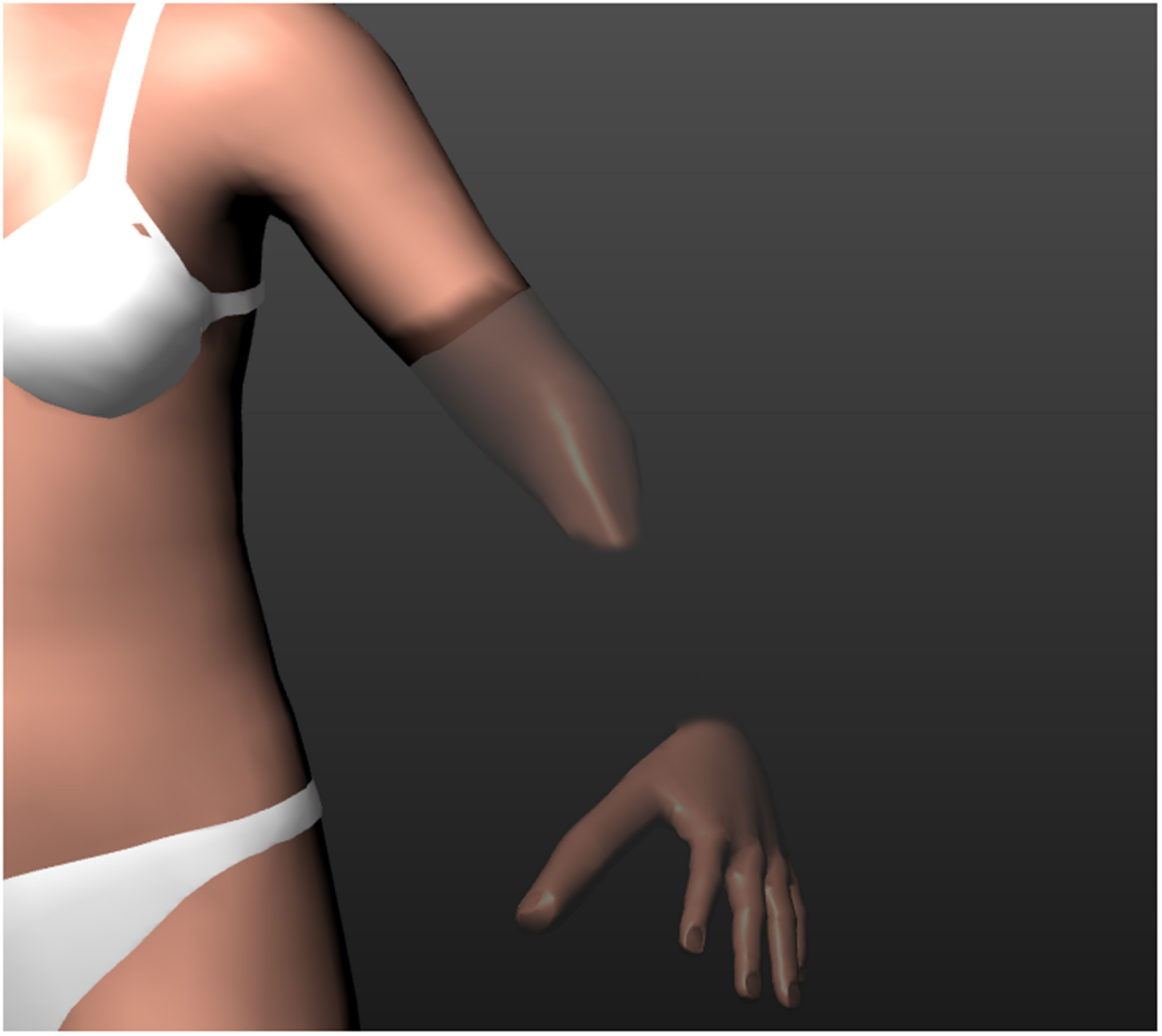
A conceptual illustration of how to visualize the residual limb and phantom limb. The “presence” of the phantom limb is indicated by its visibility, meaning that the invisible parts are no longer perceived by the patient.

The distinction between PLP and residual limb pain could be simplified in a future version of C.A.L.A. by a clear visual differentiation between residual limb and phantom limb in the representation of the 3D avatar (see Figure 9). This would make it more obvious, both when drawing and when evaluating pain, whether the pain is located at the stump or actual PLP is experienced. Considering that pain in general is probably the most important aspect of quality-of-life-limiting discomfort, we consider it useful to expand C.A.L.A. to include the documentation of stump pain as well.

As described in the literature (2, 20) and also observed in some patients, phantom sensation was not present in the entire lost limb, but only in the distal areas of the phantom. To increase the precision of the representation, this circumstance could be represented by masking the areas of the phantom that are no longer perceived (see Figure 9).

### Adjusting the Functionality

In addition to these two main topics, we have identified several other contexts in which C.A.L.A. could be improved to increase its usability and validity. The most relevant ones are listed below.

When adjusting the position of the phantom limb, the 3D avatar is initially in a position where arms and fingers are slightly spread and bent. While this body position is advantageous for painting and deforming the phantom, we think that a more standardized body position, such as the neutral-zero position (27), would be more beneficial for phantom limb positioning. We believe that such an alignment of the initial position will not only facilitate the positioning of the phantom, but will also increase the significance of the quantified position. The range of functions concerning the positioning and deformation of the phantom has shown that the currently provided options can only partially cover the large variety of different perceptions. Especially for hand and fingers, but also for foot and toes, it would be required to allow adjusting them in further detail.

Another feature that has been mentioned several times was the desire for a visual representation of the progress of the phantom over the course of treatment; or, in other words, over the course of several documentations. This could especially help both to clearly demonstrate the progress of therapy and to motivate the patients to continue.

Finally, participants also considered other possible application scenarios in which C.A.L.A. could be used with modified functionality. Often mentioned was the application in Complex Regional Pain Syndrome (CRPS) or stroke patients, as well as for all other situations in which patients experience an altered body image.

## Conclusions

We have created a tool that allows for the visualization and documentation of PLP and PLS. Thus, it provides a standardized form for their presentation and can be used as a descriptive and quantitative documentation method.

Based on the evaluation with the therapists, a great demand for our tool could be determined, therefore a further development of C.A.L.A. is reasonable and can contribute to increase its usability and efficiency in operation. For such an improved version, the most important additional features in our point of view are briefly listed here again: (1) introduction of pain qualities; (2) clear distinction between phantom and residual limb; (3) additional documentation of residual limb pain; (4) more precise adjustment of shape and position of individual fingers; and (5) a visualization of the course of treatment over several sessions.

C.A.L.A. can help to bridge the gap between the therapist's conception and the patient's perception of the phantom limb. The possibility to quantify the representation of the phantom offers a previously unavailable option to monitor and analyze its change over the course of treatment and can help to create insights into the correlation between certain forms of treatment and PLS or PLP. Finally, C.A.L.A. enables a more integrated representation of the phantom than is possible with conventional visualization methods with little effort regarding time and other resources, increasing feasibility regarding clinical context.

## Data Availability Statement

The raw data supporting the conclusions of this article will be made available by the authors, without undue reservation.

## Ethics Statement

The studies involving human participants were reviewed and approved by Ethics Committee of the Medical Faculty of the Eberhard-Karls-University Tuebingen. The patients/participants provided their written informed consent to participate in this study.

## Author Contributions

MB designed and implemented the first version of C.A.L.A., conceived and performed the evaluation, analyzed the data, and wrote the manuscript. JM contributed to the design of C.A.L.A., contributed to planning and performing of the evaluation, and revised the manuscript. JH provided feedback on and verified the data analysis and revised the manuscript. MVB contributed to the interpretation of the results and revised the manuscript. AD and JK contributed to critical revision of the intellectual content and approved the final version. CP conceived the original idea of the project, contributed to the design of C.A.L.A., contributed to data acquisition, revised the manuscript, and was in charge of overall direction and planning. All authors provided critical feedback and helped shape the research, analysis, and manuscript. All authors contributed to the article and approved the submitted version.

## Funding

We acknowledge support by the Open Access Publishing Fund of the University of Tuebingen.

## Conflict of Interest

The authors declare that the research was conducted in the absence of any commercial or financial relationships that could be construed as a potential conflict of interest.

## Publisher's Note

All claims expressed in this article are solely those of the authors and do not necessarily represent those of their affiliated organizations, or those of the publisher, the editors and the reviewers. Any product that may be evaluated in this article, or claim that may be made by its manufacturer, is not guaranteed or endorsed by the publisher.

## Supplementary Material

A supplementary video explaining and illustrating the functionality of C.A.L.A. can be found here: https://www.playbionic.org/cala/.
