# Supplementary material for: Visualizing the Unseen: Illustrating and Documenting Phantom Limb Sensations and Phantom Limb Pain With C.A.L.A
Source: Front Rehabil Sci. 2022 Feb 9;3:806114. doi: 10.3389/fresc.2022.806114 (PMC9397903; doi:10.3389/fresc.2022.806114)
Supplement: Supplementary file 1 [file Data_Sheet_1.PDF]

Patient Questionnaire – C.A.L.A. Phase 1

1a.) How accurately was your phantom limb pain mapped in C.A.L.A.?

Very inaccurate				Very accurate
☐	☐	☐	☐	☐
1	2	3	4	5

1b.) Could all aspects of phantom limb pain be mapped in C.A.L.A.?

☐ Yes ☐ No ☐ I don't know ☐ No Phantom Limb Pain

1c.) If no, which aspects of phantom limb pain could not be mapped?

____

____

____

2a.) How accurately was the deformation (changes in length and circumference) of your phantom mapped in C.A.L.A.?

Very inaccurate				Very accurate
☐	☐	☐	☐	☐
1	2	3	4	5

2b.) Could all aspects of deformation be mapped in C.A.L.A.?

☐ Yes ☐ No ☐ I don't know ☐ No Deformation

2c.) If no, which aspects of deformation could not be mapped?

____

____

____

3a.) How accurately was the position of your phantom mapped in C.A.L.A.?

Very inaccurate				Very accurate
☐	☐	☐	☐	☐
1	2	3	4	5

3b.) Could all aspects of position be mapped in C.A.L.A.

☐ Yes ☐ No ☐ I don't know ☐ No particular Position

3c.) If no, which aspects of position could not be mapped?

____

____

____

4.) How accurately was your body image mapped in C.A.L.A.?

Very inaccurate | Very accurate

1	2	3	4	5

5.) How accurately was your phantom limb mapped in C.A.L.A.?

Very inaccurate | Very accurate

					☐ No Phantom Limb Sensation
1	2	3	4	5	

6.) How important was it for you to adjust the physical shape when depicting your body image?

Very unimportant | Very important

1	2	3	4	5

7.) How important was it for you to adjust the age when depicting your body image?

Very unimportant | Very important

1	2	3	4	5

8.) How important was it for you to adjust the gender when depicting your body image?

Very unimportant | Very important

1	2	3	4	5

9.) Was your body image affected positively or negatively by depicting the phantom in C.A.L.A.?

☐ Positively ☐ Negatively ☐ Was not affected ☐ I don’t know

10.) Further notices

____

____

____

____

____

____
