# Supplementary material for: Visualizing the Unseen: Illustrating and Documenting Phantom Limb Sensations and Phantom Limb Pain With C.A.L.A
Source: Front Rehabil Sci. 2022 Feb 9;3:806114. doi: 10.3389/fresc.2022.806114 (PMC9397903; doi:10.3389/fresc.2022.806114)
Supplement: Supplementary file 2 [file Data_Sheet_2.PDF]

Patienten-Fragebogen – C.A.L.A. Phase 1

1a.) Wie genau wurden Ihre Phantomschmerzen in C.A.L.A. erfasst?

Sehr ungenau — Sehr genau

1	2	3	4	5

1b.) Konnten alle Aspekte der Phantomschmerzen in C.A.L.A. erfasst werden?

☐ Ja ☐ Nein ☐ Weiß nicht ☐ Kein Phantomschmerz

1c.) Wenn nein, welche Aspekte der Phantomschmerzen konnten nicht erfasst werden?

____

____

____

2a.) Wie genau wurde die Deformierung (Veränderung in Länge und Umfang) Ihres Phantoms in C.A.L.A. erfasst?

Sehr ungenau — Sehr genau

1	2	3	4	5

2b.) Konnten alle Aspekte der Deformierung in C.A.L.A. erfasst werden?

☐ Ja ☐ Nein ☐ Weiß nicht ☐ Keine Deformierung

2c.) Wenn nein, welche Aspekte der Deformierung konnten nicht erfasst werden?

____

____

____

3a.) Wie genau wurde die Positionierung/Haltung/Stellung Ihres Phantoms in C.A.L.A. erfasst?

Sehr ungenau — Sehr genau

1	2	3	4	5

3b.) Konnten alle Aspekte der Positionierung/Haltung/Stellung in C.A.L.A. erfasst werden?

☐ Ja ☐ Nein ☐ Weiß nicht ☐ Keine besondere Stellung

3c.) Wenn nein, welche Aspekte der Positionierung/Haltung/Stellung konnten nicht erfasst werden?

____

____

____

4.) Wie genau wurde Ihr Körperbild nach Ihrem Empfinden in C.A.L.A. abgebildet?

Sehr ungenau — Sehr genau

1	2	3	4	5

5.) Wie genau wurde Ihr Phantom nach Ihrem Empfinden in C.A.L.A. abgebildet?

Sehr ungenau — Sehr genau

					☐ Kein Phantomempfinden
1	2	3	4	5	

6.) Wie wichtig war Ihnen beim Abbilden des Körperbildes das Anpassen des Gewichtes/der Konstitution?

Sehr unwichtig — Sehr wichtig

1	2	3	4	5

7.) Wie wichtig war Ihnen beim Abbilden des Körperbildes das Anpassen des Alters?

Sehr unwichtig — Sehr wichtig

1	2	3	4	5

8.) Wie wichtig war Ihnen beim Abbilden des Körperbildes das Anpassen des Geschlechtes (m/w/d)?

Sehr unwichtig — Sehr wichtig

1	2	3	4	5

9.) Wurde Ihr Körperbild durch das Abbilden des Phantoms in C.A.L.A. positiv oder negativ beeinflusst?

☐ Positiv ☐ Negativ ☐ Neutral ☐ Weiß nicht

10.) Weitere Anmerkungen zum Patienten

____

____

____

____

____

____
