# Supplementary material for: Visualizing the Unseen: Illustrating and Documenting Phantom Limb Sensations and Phantom Limb Pain With C.A.L.A
Source: Front Rehabil Sci. 2022 Feb 9;3:806114. doi: 10.3389/fresc.2022.806114 (PMC9397903; doi:10.3389/fresc.2022.806114)
Supplement: Supplementary file 3 [file Data_Sheet_3.PDF]

Therapist Questionnaire C.A.L.A.

1. Do you use specific documentation templates when assessing your patients?
(Multiple answers possible)

☐ I use body charts printed on paper, as shown here, and draw abnormalities on them.

☐ I make a written note of what the patient gives me feedback on.

☐ Others:

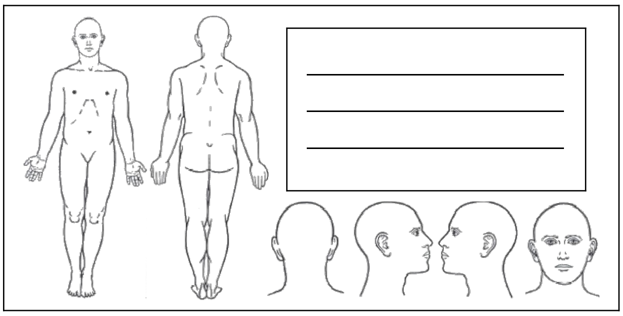

____

____

____

____

2. What parameters are important to you in the assessment of findings in relation to the patient's body image?

	Not important 1	2	3	4	Very important 5
a) Height, weight, BMI					
b) Constitution / physique					
c) Statics / Posture					
d) Circumferential dimensions					
e) Length dimensions					
f) Phantom Pain					
g) Stump pain					
h) Sensory disturbances					
j) Muscle tension disorders					

k) Others:

____

____

____

3. How do you record your patients' pain? (Multiple answers possible)

- ☐ Numeric Rating Scale (NRS 0-10)
- ☐ Visual Analogue Scale (VAS)
- ☐ Pain Detect
- ☐ German Pain Questionnaire
- ☐ Pain Disability Index
- ☐ MPI-D
- ☐ Others:

____

____

____

4. What aspects of pain do you record during the assessment?

	Not important 1	2	3	4	Very important 5
a) Pain intensity at rest					
b) Pain intensity during movement					
c) Pain intensity during stress					
d) Pain localization					
e) Pain quality					

f) Others:

____

____

____

____

5. How many amputation patients (upper/lower extremity) have you treated in the past 6 months?

☐ 1-5 Patients ☐ 6-10 Patients ☐ 11-15 Patients ☐ 16-20 Patients ☐ > 20 Patients

6. Over what period of time do you treat these amputation patients?

☐ < 1 Month ☐ 1-2 Months ☐ 3-4 Months ☐ 5-6 Months ☐ > ½ Year

7. With what weekly frequency do you treat these amputation patients?

☐ 1x/Week ☐ 2x/Week ☐ 3x/Week ☐ 4x/Week ☐ daily

8. Do you have the technical requirements in your office to use C.A.L.A.?

☐ Yes, I have a laptop/PC with Windows 10 on which I can install software myself (user with admin rights)

☐ No, I need a laptop

9. How do you assess the diagnostic value of C.A.L.A.?

Very low — Very high

1	2	3	4	5

10. How do you assess the therapeutic value of C.A.L.A.?

Very low — Very high

1	2	3	4	5

11. How helpful would you rate C.A.L.A. In the following respects compared to your previous documentation method?

	Not helpful 1	2	3	4	Very helpful 5
a) Modeling the phantom limb					
b) Positioning the phantom limb					
c) Drawing pain and cramps onto the phantom limb					
d) Visualization of the body image					
e) Quantification of body measurements					
f) Quantification of pain and cramps					

12. How can we make C.A.L.A. easier to operate/use?

____

____

____

____

13. What content features/parameters are you missing in C.A.L.A.?

____

____

____

____

14. Can you imagine using C.A.L.A. for phantom limb pain therapy as well? If so, what other functions would you need for this?

____

____

____

____

15. Can you imagine using C.A.L.A. also for the diagnosis/therapy of CRPS patients? If yes, which functions would you still need for this?

____

____

____

____

16. Can you think of other patient diagnoses besides CRPS where you (and possibly the patients) would benefit from C.A.L.A. application?

____

____

____

____
