# Supplementary material for: Visualizing the Unseen: Illustrating and Documenting Phantom Limb Sensations and Phantom Limb Pain With C.A.L.A
Source: Front Rehabil Sci. 2022 Feb 9;3:806114. doi: 10.3389/fresc.2022.806114 (PMC9397903; doi:10.3389/fresc.2022.806114)
Supplement: Supplementary file 4 [file Data_Sheet_4.PDF]

Therapeuten-Fragebogen C.A.L.A.

1. Verwenden Sie bei der Befunderhebung Ihrer Patienten bestimmte Dokumentationsvorlagen?
(Mehrfachnennungen möglich)

☐ Ich verwende, wie hier dargestellt, auf Papier gedruckte Bodycharts und zeichne darin Auffälligkeiten ein.

☐ Ich notiere schriftlich, was mir der Patient rückmeldet.

☐ Sonstiges:

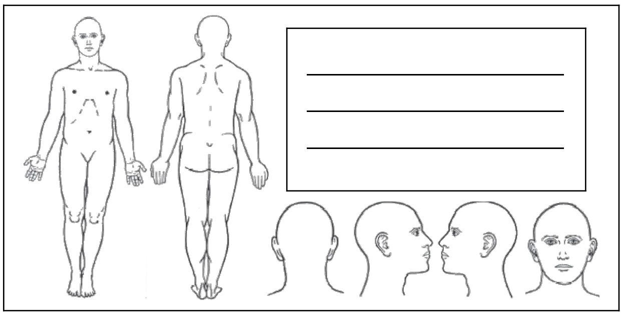

____

____

____

____

2. Welche Parameter sind Ihnen bei der Befunderhebung <u>im Bezug auf das Körperbild</u> der Patienten wichtig?

Nicht wichtig — Sehr wichtig

	1	2	3	4	5
a) Größe, Gewicht, BMI					
b) Konstitution / Körperbau					
c) Statik / Körperhaltung					
d) Umfangsmaße					
e) Längenmaße					
f) Phantomschmerzen					
g) Stumpfschmerzen					
h) Sensibilitätsstörungen					
j) Muskelspannungsstörungen					

k) Sonstige:

____

____

____

3. Wie erfassen Sie die Schmerzen Ihrer Patienten? **(Mehrfachnennungen möglich)**

- ☐ Numeric Rating Scala (NRS 0-10)
- ☐ Visuell Analoge Skala (VAS)
- ☐ Pain Detect
- ☐ Deutscher Schmerzfragebogen
- ☐ Pain Disability Index
- ☐ MPI-D
- ☐ Sonstige:

____

____

____

4. Welche Aspekte von Schmerzen erfassen Sie bei der Befunderhebung?

Nicht wichtig – Sehr wichtig

	1	2	3	4	5
a) Schmerzintensität in Ruhe					
b) Schmerzintensität bei Bewegung					
c) Schmerzintensität bei Belastung					
d) Schmerzlokalisation					
e) Schmerzqualität					

f) Sonstige:

____

____

____

____

5. Wie viele Amputationspatienten (obere/untere Extremität) haben Sie in den vergangenen 6 Monaten behandelt?

☐ 1-5 Patienten ☐ 6-10 Patienten ☐ 11-15 Patienten ☐ 16-20 Patienten ☐ > 20 Patienten

6. Über welchen Zeitraum behandeln Sie diese Amputationspatienten?

☐ < 1 Monat ☐ 1-2 Monate ☐ 3-4 Monate ☐ 5-6 Monate ☐ > ½ Jahr

7. Mit welcher wöchentlichen Häufigkeit behandeln Sie diese Amputationspatienten?

☐ 1x/Woche ☐ 2x/Woche ☐ 3x/Woche ☐ 4x/Woche ☐ täglich

8. Verfügen Sie in Ihrer Praxis über die technischen Voraussetzungen, um Cala zu benutzen?

☐ Ja, ich verfüge über einen Laptop/PC mit Windows 10, auf dem ich selbst Software installieren kann (Benutzer mit Admin-Rechten)

☐ Nein, ich benötige einen Laptop

9. Wie schätzen Sie den diagnostischen Wert von C.A.L.A. ein?

Sehr niedrig — Sehr hoch

☐	☐	☐	☐	☐
1	2	3	4	5

10. Wie schätzen Sie den therapeutischen Wert von C.A.L.A. ein?

Sehr niedrig — Sehr hoch

☐	☐	☐	☐	☐
1	2	3	4	5

11. Wie hilfreich schätzen Sie C.A.L.A. In den folgenden Punkten im Vergleich zu Ihrer bisherigen Dokumentationsmethode ein?

	Nicht hilfreich 1	2	3	4	Sehr hilfreich 5
a) Modellierung des Phantoms	☐	☐	☐	☐	☐
b) Positionierung des Phantoms	☐	☐	☐	☐	☐
c) Einzeichnen von Schmerzen und Verkrampfungen am Phantom	☐	☐	☐	☐	☐
d) Visualisierung des Körperbildes	☐	☐	☐	☐	☐
e) Quantifizierung der Körpermaße	☐	☐	☐	☐	☐
f) Quantifizierung der Schmerzen und Verkrampfungen	☐	☐	☐	☐	☐

12. Wie können wir die Bedienung/Benutzung von C.A.L.A. erleichtern?

____

____

____

____

13. Welche inhaltlichen Funktionen/Parameter fehlen Ihnen in C.A.L.A.?

____

____

____

____

14. Können Sie sich vorstellen, C.A.L.A. auch für die Therapie von Phantomschmerzen zu verwenden? Wenn ja, welche Funktionen würden Sie dazu noch benötigen?

____

____

____

____

15. Können Sie sich vorstellen, C.A.L.A. auch für die Diagnose /Therapie von CRPS Patienten zu verwenden? Wenn ja, welche Funktionen würden Sie dazu noch benötigen?

____

____

____

____

16. Fallen Ihnen außer CRPS noch andere Patienten-Diagnosen ein, bei denen Sie (und möglicherweise auch die Patienten) von der C.A.L.A. Anwendung profitieren würden?

____

____

____

____
