# Supplementary material for: Visualizing the Unseen: Illustrating and Documenting Phantom Limb Sensations and Phantom Limb Pain With C.A.L.A
Source: Front Rehabil Sci. 2022 Feb 9;3:806114. doi: 10.3389/fresc.2022.806114 (PMC9397903; doi:10.3389/fresc.2022.806114)
Supplement: Supplementary file 5 [file Data_Sheet_5.PDF]

# C.A.L.A. - Study Phase 1
# Case descriptions

## Case description 1:

### == Base Model 1 ==

- Male, 27 years
- weight: approx. 74 kg
- normal muscular
- slim constitution
- height: 180cm
- upper arm: circumference 20.9 cm, length 25.4 cm
- forearm: circumference 22.3 cm, length 14.8 cm

### == Case 1: ==

Transhumeral amputation left

Pain

- Upper arm medial: slight
- Forearm circular: moderate
- Hand palmar: extreme

Cramping

- Forearm palmar: slight
- Finger & hand palmar: strong

Modeling the phantom

- Forearm shortened to half length (telescoping)
- Fingers are perceived as very thin

Positioning the phantom

- shoulder joint: slight flexion and abduction, distinct internal rotation
- elbow: 90° flexion, pronated
- wrist: palmar flexion
- finger: fist

# C.A.L.A. - Study Phase 1
# Case descriptions

## Case description 2:

### == Base Model 2 ==

- Female, 55 years
- Weight: approx. 95 kg
- clearly overweight
- height: 165 cm
- thigh: circumference 69 cm, length 35.8 cm
- lower leg: circumference 48.4 cm, length 37 cm

### == Case 2: ==

Transfemoral amputation right

Pain

- Upper leg distal, circular: moderate pain
- Lower leg medial: severe pain
- Ankle circular: mild pain

Cramping

- Foot whole inside: medium

Modeling the phantom

- Lower leg: length felt shortened to approx. 1/3, is felt clearly enlarged
- Foot: is felt enlarged

Positioning the phantom

- Hip joint: normal position
- Knee: rotated outwards (approx. 160°)
- Foot: maximum inversion, slight dorsal extension
- Big toe: maximum hyperextension
