# Supplementary material for: Visualizing the Unseen: Illustrating and Documenting Phantom Limb Sensations and Phantom Limb Pain With C.A.L.A
Source: Front Rehabil Sci. 2022 Feb 9;3:806114. doi: 10.3389/fresc.2022.806114 (PMC9397903; doi:10.3389/fresc.2022.806114)
Supplement: Supplementary file 6 [file Data_Sheet_6.PDF]

C.A.L.A. - Studie Phase 1
Fallbeschreibungen

Fallbeschreibung 1:

== Grundmodell 1 ==
- Männlich, 27 Jahre
- Gewicht: ca. 74 kg
- normal muskulär
- schlanke Konstitution
- Körpergröße: 180cm
- Oberarm: Umfang 20.9 cm, Länge 25.4 cm
- Unterarm: Umfang 22.3 cm, Länge 14.8 cm

== Fall 1: ==
Transhumerale Amputation links

Schmerzen
- Oberarm medial: gering
- Unterarm zirkulär: mittelstark
- Hand palmar: extrem

Verkrampfung
- Unterarm palmar: leicht
- Finger & Hand palmar: stark

Modellierung Phantom
- Unterarm auf halbe Länge verkürzt (Telescoping)
- Finger werden als sehr dünn empfunden

Positionierung Phantom
1. Schultergelenk: leichte Flexion und Abduktion, deutliche Innenrotation
2. Ellenbogen: 90° gebeugt, proniert
3. Handgelenk: Palmar Flexion
4. Finger: Faust

C.A.L.A. - Studie Phase 1
Fallbeschreibungen

Fallbeschreibung 2:

== Grundmodell 2 ==
- Weiblich, 55 Jahre
- Gewicht: ca. 95 kg
- deutlich übergewichtig
- Körpergröße: 165 cm
- Oberschenkel: Umfang 69 cm, Länge 35.8 cm
- Unterschenkel: Umfang 48.4 cm, Länge 37 cm

== Fall 2: ==
Transfemorale Amputation rechts

Schmerzen
- Obeschenkel distal, zirkulär: mittlerer Schmerz
- Unterschenkel medial: starker Schmerz
- Knöchel zirkulär: leichter Schmerz

Verkrampfung
- Fuß ganze Innenseite: mittelstark

Phantom Modellierung
- Unterschenkel: Länge gefühlt verkürzt auf ca. 1/3, wird deutlich vergrößert empfunden
- Fuß: wird vergrößert empfunden

Phantom Position
- Hüftgelenk: normale Stellung
- Knie: nach außen rotiert (ca. 160°)
- Fuß: maximale Inversion, leichte Dorsal Extension
- Großer Zeh: maximal überstreckt
